# Microbial Group Dynamics in Plant Rhizospheres and Their Implications on Nutrient Cycling

**DOI:** 10.3389/fmicb.2018.01516

**Published:** 2018-07-11

**Authors:** Joshua Garcia, Jenny Kao-Kniffin

**Affiliations:** School of Integrative Plant Science, Cornell University, Ithaca, NY, United States

**Keywords:** holobiont, group selection, microbiome, multilevel selection, nitrogen, rhizosphere

## Abstract

Plant rhizospheres encompass a dynamic zone of interactions between microorganisms and their respective plant hosts. For decades, researchers have worked to understand how these complex interactions influence different aspects of plant growth, development, and evolution. Studies of plant-microbial interactions in the root zone have typically focused on the effect of single microbial species or strains on a plant host. These studies, however, provide only a snapshot of the complex interactions that occur in the rhizosphere, leaving researchers with a limited understanding of how the complex microbiome influences the biology of the plant host. To better understand how rhizosphere interactions influence plant growth and development, novel frameworks and research methodologies could be implemented. In this perspective, we propose applying concepts in evolutionary biology to microbiome experiments for improved understanding of group-to-group and community-level microbial interactions influencing soil nutrient cycling. We also put forth simple experimental designs utilizing -omics techniques that can reveal important changes in the rhizosphere impacting the plant host. A greater focus on the components of complexity of the microbiome and how these impact plant host biology could yield more insight into previously unexplored aspects of host-microbe biology relevant to crop production and protection.

## Introduction

With growing interest in host-microbe biology, a mixture of new and re-established terms have been developed in recent years to describe complex associations of organisms that are heritable. The concept of the “holobiont” and its “hologenome” (i.e., a host and all of its microbial symbionts, and consequently, the collective genomes of the adaptive unit) (Rosenberg and Zilber-Rosenberg, 2016) signify the potential for natural selection to act, not only on the individual, but also on its suite of associations with its microbial members. While much of the discussion on the holobiont–hologenome theory has focused on animals and their microbiomes, the concept appropriately extends to plants and their associated microbiomes.

There is an extensive history of research detailing the co-evolutionary forces dually acting on plants and their microbial symbionts. For example, research on vesicular-arbuscular mycorrhiza (VAM) has suggested that the highly beneficial symbiosis between the plant root and fungal symbiont has driven the diversification of plant root morphology as well as VAM structure and function (Brundrett, 2002). In addition, decades of legume research suggest that interactions, such as plant sanctions against nitrogen-fixing rhizobia, are largely what help stabilize the easily compromised legume-rhizobium mutualism (Masson-Boivin and Sachs, 2017). In more recent years, the advent of next-generation sequencing and other -omics tools has generated interest in better understanding how more complex associations between plants and their microbiome, termed the “phytobiome” (Leach et al., 2017), play a role in plant fitness, as well as plant health, growth, and development.

Similar to animal microbiomes, different compartments of a plant can harbor functionally and taxonomically distinct sets of microbiomes—these include the phyllosphere microbiome encompassing the aboveground parts of plants, root, and shoot endophytes (microbiomes colonizing root and shoot tissues), and the rhizosphere microbiome that inhabits soil surrounding plant roots and adhering to root surfaces (Turner et al., 2013). Although the rhizosphere is not a plant organ or a physically intact compartment of the plant, this narrow band of soil surrounding roots harbors a tremendous diversity of microorganisms that are free-living or intricately linked to their plant hosts (Mendes et al., 2013). The root zone is an environment that is heavily enriched in compounds that are secreted by both plants and microorganisms, and play a key role in maintaining plant-microbe interactions (Badri et al., 2009). The exudates include sugars, complex polysaccharides, amino acids, proteins, and a multitude of secondary metabolites (Badri et al., 2009). An important component of these compounds includes signaling of host-microbe and microbe-microbe interactions (Venturi and Keel, 2016). With hundreds of different microbial taxa inhabiting a plant’s rhizosphere, the possibilities of interactions shaping nutrient dynamics impacting plant growth are expansive. In this perspective, we discuss the background research on community evolution and genetics that are applicable to rhizosphere controls on nutrient cycling. We then propose conceptual ideas for experiments that explicitly test group-level dynamics in rhizospheres that can be targeted to alter nutrient capture and utilization processes impacting plant performance.

## A Novel Framework for Understanding Rhizosphere Microbiomes

While it appears overwhelming to study complex interactions between a microbiome and its host plant, there is growing interest in broadening the concept of heritability to be inclusive of a host and its microbiome (van Opstal and Bordenstein, 2015). It is conceivable that a plant’s rhizosphere microbiome is an ecological unit with heritable traits, although it is explicitly a physical extension of the host because it involves the soil habitat surrounding roots and not an intact unit of a plant. This concept of an “extended phenotype” is not new, but is derived from the concept proposed by Dawkins (1982) that states an organism’s phenotype should extend from its cellular components to its environment. Examples of extended phenotypes include manipulation of an organism’s physical environment and alterations to behaviors, both of which can start at the gene-level. Other concepts in evolutionary biology propose a broader view that heritability is shaped at multiple levels beyond the individual, such that natural selection acts on ecological units beyond the individual. Perhaps one of the most salient among them is the concept of multilevel selection, which is often referred to as group selection.

Multilevel selection can be defined as natural selection acting on whole groups of organisms in addition to individuals, as initially proposed by Darwin (Wilson and Wilson, 2008). During the latter half of the 20th century, many evolutionary biologists applied this concept to study the evolution of organisms, such as insects, small mammals, and humans (Wilson and Wilson, 2008). While the framework typically has been used to study animals, very little work has been done to examine how the concept may extend to plants and their associated microbiomes. Applying the concept to the plant rhizosphere could help to elucidate the complex interactions occurring in microbe-microbe and microbe-plant networks that can be acted upon by natural and artificial selection at multiple levels. The application of this concept to microbiome science may be especially worthwhile given the types of selective pressures that dictate microbial density-dependent rhizosphere processes, such as nutrient cycling.

**FIGURE 1:**
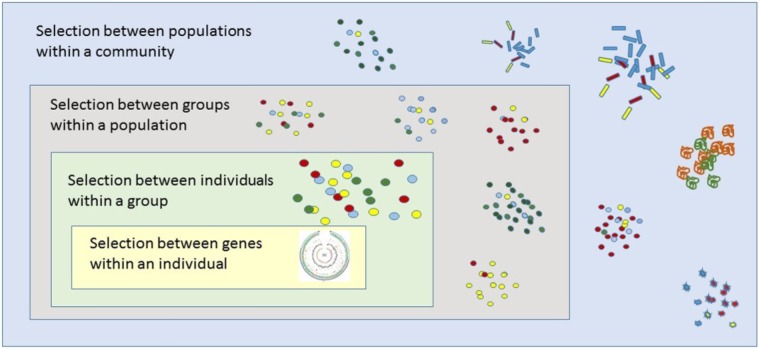
Applying group-level concepts in evolutionary biology to the study of microbiomes. A key tenant of multilevel selection theory is that selection not only acts on individuals, but can act on multiple levels of organization across biological units to influence the observed phenotype. For microbiota inhabiting rhizospheres, selection can act on different levels of organization (including simultaneously) consisting of genes, individuals, cells, groups, and the holobiont (i.e., plant host with its extended microbiome). The figure is adapted from Wilson and Wilson (2008).

A key tenant of multilevel selection theory is that selection not only acts on individuals, but can act on multiple levels of organization across biological units to influence the observed phenotype (**Figure 1**) (Wilson and Wilson, 2008). A classic example includes cooperative sharing of a common resource such as food within a group. Evolutionary biologists have long questioned how cooperative resource sharing could arise within a group, given that cooperation can lower one’s fitness according to the classic selfish-gene view (Sachs et al., 2004). In the case of resource sharing among members of a group, those who utilize the resource in an uncooperative manner, sometimes referred to as “cheaters,” can circumvent paying the price of cooperation while reaping the benefits of utilizing the resource, thereby increasing their own fitness (Riehl and Frederickson, 2016). Such a scenario could ultimately lead to a “tragedy of the commons” (Waite and Shou, 2012). Studies of microbial populations, however, have shown that groups of cooperative individuals could arise and outcompete cheaters if certain group-level selective pressures determine fitness outcomes at the population or community level (MacLean and Gudelj, 2006; Boyle et al., 2012; Allen and Nowak, 2013).

In the case of the plant rhizosphere, individual and group-level selective pressures constantly interact to shape the phenotype of the rhizosphere, although this is often overlooked when focusing on classical symbiosis studies. If perceptions of symbiosis expand to encompass multiple partnerships, including dozens of microbial taxa, it is then suitable to test the effect of group dynamics on nutrient cycling in the rhizosphere influencing plant growth. A plant growing in nitrogen-limited soils could gain a fitness advantage over competitors by enriching its rhizosphere for microbial communities that enhance nutrient capture and utilization capabilities (DeAngelis et al., 2009; Kao-Kniffin and Balser, 2010; Panke-Buisse et al., 2015).

**FIGURE 2:**
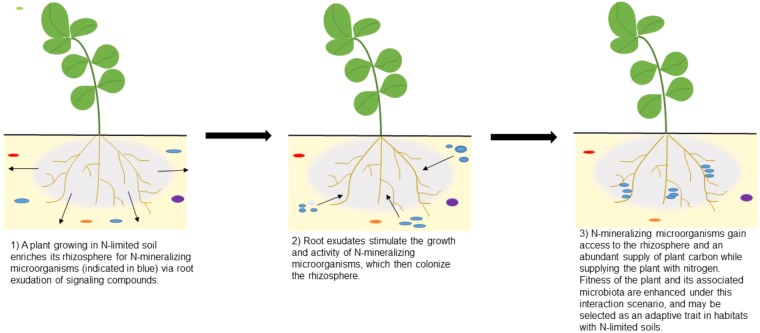
Enrichment of microbial groups in plant rhizospheres grown under nitrogen-limited soils. The illustrations depict a scenario in which bacteria form groups within the rhizosphere, with members that produce a specific extracellular enzyme (e.g., N-Acetyl glucoseaminidase, NAGase) targeting a particular substrate (e.g., chitin). Nitrogen that was bound to soil organic matter comprised of chitin is mineralized and captured by nearby microorganisms. Upon microbial cell turnover, these hotspots of inorganic nitrogen can be intercepted by actively growing plant roots. It is conceivable that plants and groups of microorganisms have formed complex associations that can be adaptive under resource limitation. Applying multilevel selection theory to microbiome studies could reveal novel cross-kingdom signaling mechanisms that challenge traditionally defined units of heritability.

For example, actively growing roots could signal for microorganisms that are capable of producing extracellular enzymes that release nitrogen bound in soil organic matter (Jacoby et al., 2017; Lemanceau et al., 2017). For prokaryotes, these mineralization processes are density-dependent and need a quorum of producers to sufficiently access key nutrients in soil (DeAngelis et al., 2008). The groups of producers are not kin-based and can be formed from taxonomically diverse microbiota if they have the ability to biosynthetically produce the specific enzymes that are secreted into the soil. In this scenario, selection could favor microhabitats in rhizospheres that promote coordinated group behaviors that enhance plant access to nitrogen or phosphorus upon cell turnover, while the microorganisms benefit from having an abundant supply of carbon and other nutrients from plant roots (**Figure 2**). While the example illustrates how group dynamics at multiple levels of organization impact the fitness of the plant host, the microbial community, and specific individuals or related cells, the interaction has posed a significant challenge to test experimentally until recent years.

There are many more components to nutrient cycling that are microbially mediated, with potential group dynamics influencing plant growth. For example, microorganisms such as rhizobia and certain free-living diazotrophs can form groups that fix atmospheric nitrogen through metabolic processes and make the nutrient readily available for plant uptake (Santi et al., 2013; Oldroyd and Dixon, 2014; Mus et al., 2016). In addition, certain microbial taxa are known to mobilize phosphorus in the soil via the production of extracellular compounds and enzymes, which increases phosphorus availability for plant use (Vessey, 2003; Adnan et al., 2017; Alori et al., 2017). Other important plant nutrients, such as iron, can be made into plant available forms by groups of unrelated microbial taxa, through the production of compounds such as siderophores (Vessey, 2003; Radzki et al., 2013). Coordinated groups of microorganisms may also enhance plant nutrient uptake through more indirect mechanisms, such as stimulating root growth via phytohormone production, which can increase plant access to essential nutrients (Adesemoye and Kloepper, 2009).

## Observing Multiple Levels of Selection in the Plant Rhizosphere

The application of multilevel selection theory to rhizosphere processes, such as nutrient cycling, offers researchers an opportunity to understand how multiple levels of selection shape a plant’s microbiome. Experimental tests of group dynamics in the rhizosphere impacting a plant phenotype are needed to help us understand the role of complex associations of microorganisms in determining plant growth, development, and fitness. In this section, we propose three concepts for experimental designs that could aid in elucidating the role of the rhizosphere microbiome in impacting various plant phenotypes via alterations in nutrient cycling processes.

### Microbial Experimental Systems and Network Analysis

Microbial experimental systems, have allowed researchers to document shifts in population structure or adaptive changes in organisms over time (Jessup et al., 2005). The experiments were typically conducted under highly controlled conditions involving paired comparisons of cultivated bacterial strains or lower eukaryotes, to reveal outcomes of interactions. While the greatest utility of microbial experimental systems is the ability to control for environmental and biological variables that mask potential microbe-microbe interactions, hierarchies of complexity can be introduced into the system to reveal more dynamic interactions. In recent years, a small number of these experimental systems were developed to evaluate how changes in complex soil microbiomes can influence the biology of a plant following generations of selection for microbiota associated with the plant trait. For example, work by Swenson et al. (2000) demonstrated that the above-ground biomass of *Arabidopsis thaliana* could be enhanced by artificially selecting for rhizosphere microbiomes that are known to increase plant biomass production. In a similar study using *A. thaliana*, flowering time was modified by soil microbiota through repeated selections of rhizosphere microbiomes, which was predicated based on the idea that selection can be performed on a plant’s phenotype while the agents of selection are comprised of the rhizosphere microbiome (Panke-Buisse et al., 2015). The experiment resulted in taxonomic and functional shifts in the microbiome across early versus delayed flowering plants, but at the time of the study, group-level behaviors were challenging to measure when studying soil microbiomes.

Since then, advances in sequencing technology and bioinformatic analysis have provided an opportunity to examine networks of associations across multiple microbial partners that may indicate coordination across taxa (Faust and Raes, 2002). An experiment can be designed to test for associations among microbial taxa relevant to group-level processes, such as mineralization dynamics in the soil. Given that certain plant community traits could be predictive of soil microbiome composition (Leff et al., 2018), plant-soil systems that show greater nutrient capture or use efficiency over time could indicate more associations among microbial taxa, including positive, negative, or neutral, that may aid in modeling complex interactions of microbial partners in a soil system. Network analysis involving soil microbiomes could indicate specific taxa that co-occur consistently in an experimental system, which could signal targets for assembling synthetic microbial communities in rhizosphere studies (Großkopf and Soyer, 2014). Top-down approaches involving the use of microbial experimental systems for generating plant trait-associated microbiomes, followed by bottom-up approaches that use the resulting sequencing and bioinformatic data to design synthetic microbial communities, could help identify consortia that influence plant growth via alterations to nutrient cycling. These consortia of isolates could then be tested in various controlled environment and field settings, which would yield insight into interactions that alter plasticity of plant host traits.

### Observing Microbiome Controls Over Observed Phenotypes of the Plant Using -Omics Techniques

Advances in sequencing technologies provide opportunities to examine how microbiomes or individual strains of microorganisms impact plant traits through changes in plant gene expression (Schenk et al., 2012). While it is widely acknowledged that soil microorganisms alter plant developmental processes (van Loon, 2007), sequencing techniques such as transcriptomics, offer researchers an opportunity to observe these alterations at previously unexplored scales (Wolf, 2013). Microbial influences on its plant host can be observed at the gene level using plant transcriptional analysis methods, and then combined with network analysis to indicate individual genes or entire metabolic pathways that are impacted when a specific microbial partner or consortia of partners interacts with its host. Experiments can be designed to observe the effect of specific microorganisms on the development of plant traits, while observing changes in a host’s transcriptome over time. From these experiments, the effect of specific microbial associations in the rhizosphere, including effects relevant to plant nutrition requirements, could be determined.

In addition to plant transcriptomics, other -omics techniques could be applied to plant-soil systems to better understand how rhizosphere microbiomes influence host biology under different conditions. For example, exometabolomics techniques could be used to characterize microbial metabolite utilization in the rhizosphere environment (Swenson et al., 2018). This data could then be used to determine microbial exometabolic niches in the rhizosphere, which may help link metabolite composition to microbial community structure (Baran et al., 2015). Other techniques, such as proteomics, could be applied to the microbiome studies to determine how different functions of the microbiome influence the plant host (White et al., 2017). By integrating these multiple -omics techniques, researchers can develop a more robust understanding of how rhizosphere microorganisms influence soil processes and plant host biology (White et al., 2017).

### Genome-Editing Techniques to Uncover Plant Host Controls Over Microbiome Composition and Function

The development of genome-editing tools such as clustered regulatory interspaced short palindromic repeats (CRISPR)-Cas can be useful in modifying rhizosphere processes through changes in plant structure and physiology (Bortesia and Fischerab, 2015). The CRISPR-Cas method works by using short RNA sequences to guide a Cas enzyme to a target site for alteration (Nemudryi et al., 2014). Systematic alterations in plant architecture or physiology could aid in mechanistic studies of how plants control microbiome assembly and function in the rhizosphere. For example, disruption of the mutualistic interactions between microorganisms that aid in nutrient capture and plants that provide carbon sources for microbial growth could be designed through modification of root exudation processes via genetic engineering and gene-editing (Ahkami et al., 2017). Plants that no longer provide readily available carbon sources to soil microorganisms involved in mineralization dynamics may show a distinct microbial community that specializes in more complex polysaccharides found in soil organic matter. In addition, root exudation processes could be modified to produce specific families of compounds in the rhizosphere. Taxonomic shifts in the rhizosphere community could then be documented with these shifts in root exudate composition, revealing plant selection for specific microbial taxa. Additionally, researchers could study plant selection for functionally distinct microbial taxa by altering host physiology via transcription factors, similar to the methods used by Su et al. (2015). These alterations could cause functional and taxonomic shifts in the rhizosphere, which could help further our understanding of selection for specific groups of microorganisms.

## Concluding Remarks and Future Directions

Currently, there is a growing interest in developing a broader understanding of host-microbe biology. Similar to human and other animals, different plant compartments harbor distinct microbiomes, which could evolve and adapt with their host to influence the observed phenotypes. In essence, fitness is both influenced by and shared among multiple levels—the individual plant host and groups comprising its microbiome. Thus, the application of evolutionary frameworks, such as multilevel selection, to plant microbiomes could be useful in developing a more robust understanding of host-microbe interactions. Utilizing –omics techniques is key to uncovering potential mechanisms underlying group-level interactions in the rhizosphere. It is conceivable that cross-kingdom signaling dominates rhizosphere processes, which suggests that the definition of heritability should be inclusive of an individual and its associated microbiome. The plant host may influence the composition and function of the resulting microbiome, but microorganisms have the ability to modify plant traits.

An applied outcome of studying group-level dynamics in the rhizosphere is the ability to incorporate concepts of the holobiont into plant breeding. Selection efforts that consider rhizosphere microbiomes as an extended phenotype of a plant could help identify potential mechanisms that enrich for subpopulations of the microbiome. These “plant-cultivated” members of the rhizosphere could play essential roles in supporting the development of specific phenotypes of the plant that improve plant growth under biotic or abiotic stress.

However, plant genetics may not play a significant role in influencing the microbiome, but instead the plant may be highly susceptible to microbiome effects on plant traits. A greater understanding of how microbiomes influence the observed phenotype of a plant can help to tease apart the effect of environmental variables from biotic factors. For example, in studies involving genotype by environment interactions (G × E), phenotypic variation is assumed to be a result of plant genetics influenced by varying environmental conditions across field sites. In the near future, we expect that the low cost of microbiome sequencing methods will result in the adoption of rapid microbiome diagnostics revealing the role of microbiome variability across field sites in influencing plasticity of the plant phenotypes. In essence, there will likely be a shift toward analysis of genotype by environment by microbiome interactions (G × E × M) in the coming years.

Finally, we believe that the current industry focus on examining single microbial isolate effects on plant traits will be replaced with more emphasis on complex interactions involving multiple players. The recent popularity of examining synthetic communities comprised of multiple microbial strains helps to advance microbiome science forward, but it would be beneficial to move beyond cultivation-dependent methods. Applying selective filters to reduce the diversity of complex microbiomes associated with a plant trait could enable more top-down and bottom-up approaches comprised of cultivation-dependent and –independent multi-player interaction studies. While teasing apart the complexity of the rhizosphere will be incredibly challenging, such research could ultimately help develop a better understanding of how rhizosphere microbiomes influence plant growth, development, and fitness.

## Author Contributions

Both authors listed have made direct and substantial contributions to this work and have approved it for publication.

## Conflict of Interest Statement

The authors declare that the research was conducted in the absence of any commercial or financial relationships that could be construed as a potential conflict of interest.
